# Neurovascular Dysregulation During Exercise in Type 2 Diabetes

**DOI:** 10.3389/fphys.2021.628840

**Published:** 2021-04-13

**Authors:** Ann-Katrin Grotle, Jasdeep Kaur, Audrey J. Stone, Paul J. Fadel

**Affiliations:** ^1^Department of Kinesiology, The University of Texas at Arlington, Arlington, TX, United States; ^2^Department of Kinesiology and Health Education, The University of Texas at Austin, Austin, TX, United States

**Keywords:** sympathetic nerve activity, blood pressure, blood flow, exercise pressor reflex, central command, baroreflex, functional sympatholysis

## Abstract

Emerging evidence suggests that type 2 diabetes (T2D) may impair the ability to properly adjust the circulation during exercise with augmented blood pressure (BP) and an attenuated contracting skeletal muscle blood flow (BF) response being reported. This review provides a brief overview of the current understanding of these altered exercise responses in T2D and the potential underlying mechanisms, with an emphasis on the sympathetic nervous system and its regulation during exercise. The research presented support augmented sympathetic activation, heightened BP, reduced skeletal muscle BF, and impairment in the ability to attenuate sympathetically mediated vasoconstriction (i.e., functional sympatholysis) as potential drivers of neurovascular dysregulation during exercise in T2D. Furthermore, emerging evidence supporting a contribution of the exercise pressor reflex and central command is discussed along with proposed future directions for studies in this important area of research.

## Introduction

Type 2 diabetes (T2D) negatively impacts cardiovascular health, contributing to a high risk of premature mortality (Raghavan et al., 2019). Notably, ~60% of T2D patients also have hypertension, which suggests that alterations in blood pressure (BP) control are common in this population (Colosia et al., 2013). This is all very important because more than 34 million Americans currently live with T2D, a number projected to increase nearly 50% by 2050 (Boyle et al., 2010; Prevention, 2020). Notably, the prevalence of T2D and prediabetes in young adults is also increasing (Mayer-Davis et al., 2017; Prevention, 2020), which is alarming considering that an earlier disease onset is associated with high lifetime risk of cardiovascular disease (Song, 2016). These rates are, in part, attributable to the accelerated rates of sedentary lifestyle in our society (Mayer-Davis and Costacou, 2001). Indeed, physical inactivity has been shown to be an important modifiable risk factor that contributes to the development of T2D (Bowden Davies et al., 2019; Antwi et al., 2020). Although exercise is a well-recognized tool in the management of T2D due to its many cardiometabolic benefits, accumulating evidence suggests that T2D negatively affects cardiovascular responses to exercise. The most alarming consequence is an exaggerated exercise-induced BP (Scott et al., 2008; Regensteiner et al., 2009; Pinto et al., 2014; O'Connor et al., 2015; Holwerda et al., 2016a), a response associated with a heightened risk of acute adverse cerebral- and cardiovascular events (Kurl et al., 2001; Laukkanen et al., 2006). Furthermore, several studies also report reductions in contracting skeletal muscle blood flow (BF) in T2D, which likely hinders the ability to sustain exercise contributing to the well-known exercise intolerance in this population (O'Connor et al., 2012; Reusch et al., 2013; Sacre et al., 2015; Senefeld et al., 2019). Therefore, understanding the underlying mechanisms contributing to altered cardiovascular responses to exercise in T2D is crucial to identifying strategies that can reduce the heightened cardiovascular risk in this population, while at the same time improving their ability to perform and sustain physical activity.

The sympathetic nervous system plays an integral role in controlling the cardiovascular adjustments to exercise. Increases in sympathetic nerve activity (SNA) to the heart facilitate increases in cardiac output and sympathetic outflow to periphery and viscera elicits vasoconstriction of inactive skeletal muscle and tissue beds, respectively. These actions contribute to elevations in BP while facilitating increases in BF to contracting muscles (Fisher et al., 2015). Moreover, within contracting muscles, the interaction between sympathetically mediated vasoconstrictor drive and local vasodilatory factors determines active skeletal muscle BF. In this regard, locally released vasoactive compounds attenuate sympathetically mediated vasoconstriction (i.e., functional sympatholysis) (Remensnyder et al., 1962) to further facilitate increases in active muscle BF. Several neural mechanisms work in concert to facilitate these adjustments. Feedback signals from the contracting skeletal muscle (i.e., exercise pressor reflex) and feedforward signals from higher brain centers (i.e., central command) both contribute to increase SNA during exercise (Alam and Smirk, 1937; Goodwin et al., 1972; McCloskey and Mitchell, 1972). These signals also contribute to the resetting of the arterial and cardiopulmonary baroreflex, which play important roles in modulating exercise-induced increases in BP via alterations in SNA (Scherrer et al., 1990; Fadel et al., 2001; Joyner, 2006). Thus, proper neural adjustments are essential to ensuring the appropriate sympathetic and thus cardiovascular responses to exercise.

Emerging evidence suggests that T2D impairs the ability to adjust the circulation during exercise, which is highlighted by reports of exaggerated muscle SNA (MSNA) (Holwerda et al., 2016a; Vranish et al., 2020), BP (Scott et al., 2008; Regensteiner et al., 2009; Pinto et al., 2014; O'Connor et al., 2015; Holwerda et al., 2016a), and attenuated increases in contracting skeletal muscle BF (Menon et al., 1992; Kingwell et al., 2003; Lalande et al., 2008; Mac Ananey et al., 2011; Kiely et al., 2014; O'Connor et al., 2015; Groen et al., 2019; Bock et al., 2020). Therefore, the purpose of this review is to provide a brief update on the current understanding of these altered exercise responses in T2D and the potential underlying mechanisms, with an emphasis on the sympathetic nervous system and its regulation during exercise. Furthermore, we discuss emerging evidence supporting a contribution of the exercise pressor reflex and central command along with proposed future directions for studies in this important area of research.

## Cardiovascular Responses to Exercise in Type 2 Diabetes

**Figure 1:**
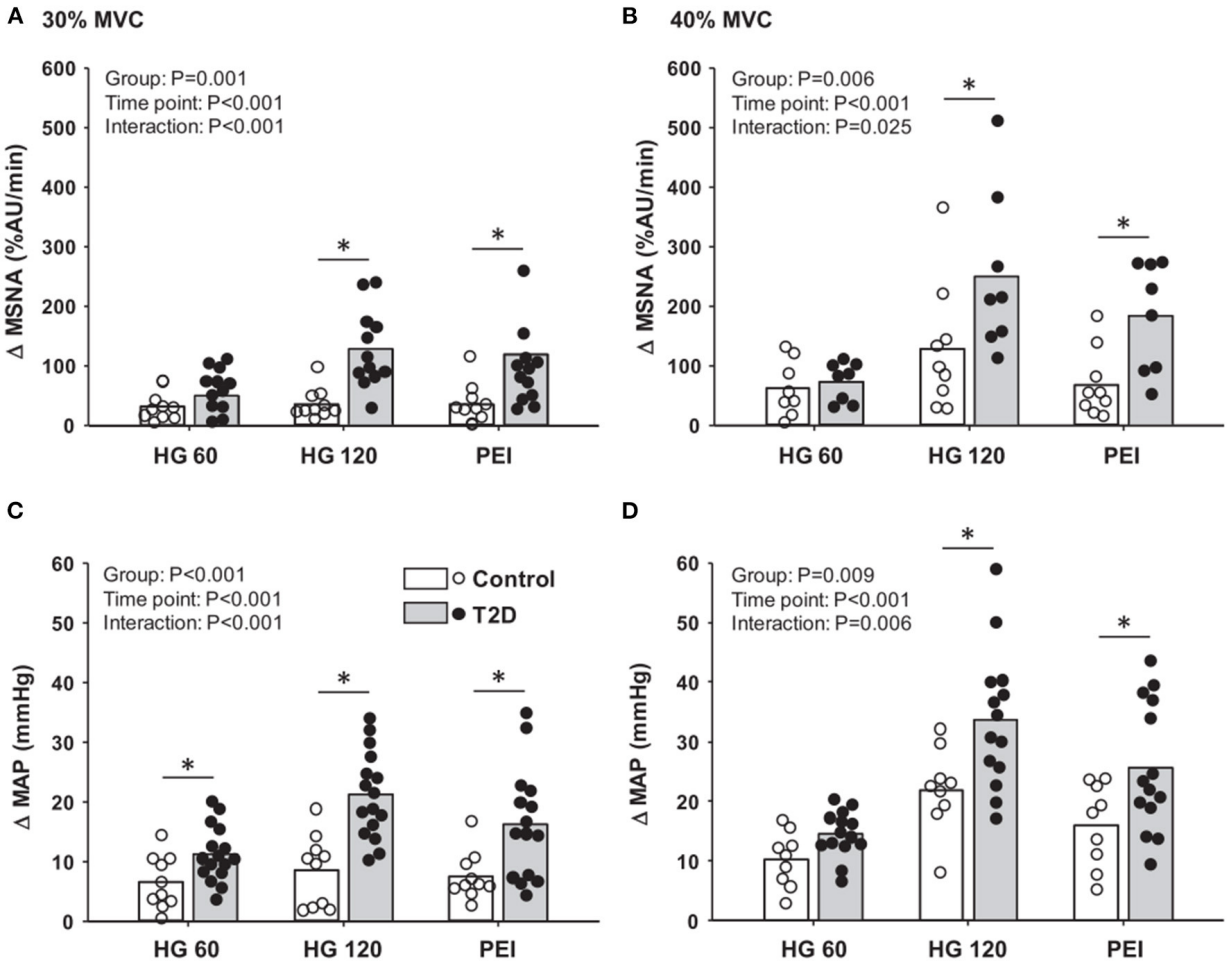
Mean data and individual data showing changes in muscle sympathetic nerve activity (MSNA; **A,B**) and mean arterial pressure (MAP; **C,D**) at 60 and 120 s of 30 and 40% maximal voluntary contraction (MVC) handgrip followed by subsequent periods of post-exercise ischemia (PEI) in type 2 diabetes (T2D) patients and control subjects. ^*^P < 0.05 vs. control. Modified from Holwerda et al. (2016a) with permission.

The first data identifying an exaggerated exercise-induced BP response in T2D patients came from studies assessing cardiovascular responses to maximal and submaximal exercise testing (Kingwell et al., 2003; Petrofsky et al., 2005; Scott et al., 2008; Regensteiner et al., 2009; Karavelioglu et al., 2013; Pinto et al., 2014; O'Connor et al., 2015). For example, Scott et al. (2008) reported an ~50% greater prevalence of an exaggerated BP response to a graded maximal treadmill test in normotensive middle-aged T2D patients relative to controls. Furthermore, an augmented BP during submaximal steady-state and short-duration constant load cycling exercise has also been reported (Andresen and Kunze, 1994; O'Connor et al., 2015). Notably, these responses were also present at an early age in which studies have shown augmented BP responses to exercise in adolescents with T2D (Pinto et al., 2014; Yardley et al., 2015). Thus, these responses highlight that T2D patients are more likely to experience exaggerated exercise-induced BP responses, which is a prognostic indicator for an augmented risk of acute myocardial infarction and stroke (Kurl et al., 2001; Laukkanen et al., 2006). A recent study by Holwerda et al. (2016a) linked the augmented BP responses to exercise in T2D with elevated SNA demonstrating, for the first time, that T2D patients, independent of coexisting hypertension, had exaggerated MSNA responses to isometric handgrip exercise compared to nondiabetic controls (Figure 1).

Another common feature reported in studies examining exercise responses in T2D has been reductions in contracting skeletal muscle BF (Menon et al., 1992; Kingwell et al., 2003; Lalande et al., 2008; Mac Ananey et al., 2011; Kiely et al., 2014; O'Connor et al., 2015; Groen et al., 2019; Bock et al., 2020). For example, seminal work by Kingwell et al. (2003) showed attenuated leg BF responses to submaximal supine cycling exercise (60% VO_2_ peak) in normotensive T2D patients. Consequently, leg vascular resistance was substantially greater in the T2D patients. Similarly, a more recent study (Groen et al., 2019) reported attenuated increases in leg BF and vascular conductance in T2D patients during single-leg knee extension exercise. However, it should be noted that conflicting findings have also been reported (Martin et al., 1995; Copp et al., 2010; Poitras et al., 2015). For example, Poitras et al. (2015) found similar increases in forearm muscle BF during a forearm critical force test in T2D patients and controls. Nonetheless, there are substantial data reporting that T2D significantly impacts the ability to properly adjust the circulation during exercise, leading to an augmented BP and reduced contracting skeletal muscle BF. For complementary details on exercise impairments in T2D, the reader is referred to other excellent reviews (Reusch et al., 2013; Green et al., 2015; Poitras et al., 2018; Kim et al., 2020; Nesti et al., 2020).

## Neurovascular Regulation During Exercise in Health

In this section, we will provide a brief overview of appropriate neurovascular regulatory mechanisms during exercise to set the stage for future sections on what is known about the potential impairments in neurovascular control mechanisms in T2D. Several neural mechanisms work in concert to regulate BP and adjust the circulation during exercise. The exercise pressor reflex is a feedback mechanism that responds to mechanical (i.e., mechanoreflex) and metabolic (i.e., metaboreflex) stimuli produced by the contracting skeletal muscle and reflexively increases MSNA, BP, HR, and respiration (Alam and Smirk, 1937; McCloskey and Mitchell, 1972; Strange et al., 1993). The afferent arm of this reflex is comprised of thinly myelinated group III (predominantly mechanically sensitive) and unmyelinated group IV (predominantly metabolically sensitive) muscle afferents (McCloskey and Mitchell, 1972; Kaufman et al., 1983). However, these afferents exhibit polymodal characteristics (Rotto and Kaufman, 1988; Rotto et al., 1990). Central command is a feedforward mechanism referring to descending signals originating from higher brain areas that simultaneously increase motor efferent drive and autonomic neural outflow that, in turn, contributes to the cardiorespiratory responses to exercise (Goodwin et al., 1972; Eldridge et al., 1981, 1985). Furthermore, both the exercise pressor reflex and central command contribute to the resetting of the arterial and cardiopulmonary baroreflex (Bevegard and Shepherd, 1966; Papelier et al., 1994; Gallagher et al., 2006). The arterial baroreflex is a negative feedback mechanism that modulates BP at rest and during exercise by making rapid cardiovascular adjustments in response to beat-to-beat changes in BP. On the other hand, the cardiopulmonary baroreflex responds to changes in central pressure and volume by reflexively adjusting MSNA at rest and during exercise (Ray et al., 1993; Ogoh et al., 2007). These neural mechanisms all converge centrally in the nucleus tractus solitarius of the medulla oblongata (Andresen and Kunze, 1994; Potts et al., 2002) and ultimately adjust sympathetic outflow via neurons in the rostral ventral lateral medulla. It is worth noting that skeletal muscle afferents also increase SNA via direct projections to the rostral ventral lateral medulla (Potts, 2006). For more in-depth discussions on neurovascular control, we refer the reader to several comprehensive reviews (Fadel and Raven, 2012; Fisher et al., 2015; Holwerda et al., 2015; Michelini et al., 2015; Nyberg et al., 2015; Grotle et al., 2020).

The resultant increases in SNA directed to visceral and peripheral blood vessels have powerful vasoconstrictor and BP-raising effects during exercise (Fairfax et al., 2013). Indeed, sympathetic vasoconstriction of inactive tissue and skeletal muscle vascular beds increases to effectively redistribute cardiac output to active skeletal muscle (Joyner et al., 1992; Rowell, 1997; Saltin et al., 1998). At the same time, sympathetically mediated vasoconstriction is attenuated in active skeletal muscle beds by local vasoactive compounds (i.e., functional sympatholysis) (Remensnyder et al., 1962) to further facilitate increases in contracting skeletal muscle BF. Although extensively studied, the sympatholytic compounds responsible for functional sympatholysis and their mechanism(s) of action are not well understood. Nevertheless, accumulating evidence supports a significant contribution played by ATP (Rosenmeier et al., 2004; Saltin and Mortensen, 2012), which appears to mediate its effect, in part, by attenuating the sensitivity of α-adrenergic receptors (Mortensen et al., 2009). Although less clear, NO also appears to contribute (Thomas and Victor, 1998), but its role may depend on the presence of other compounds such as prostacyclin (Dinenno and Joyner, 2004; Mortensen et al., 2007). Regardless, numerous studies demonstrate the presence of functional sympatholysis and its importance for increasing contracting skeletal muscle BF. Of note, functional sympatholysis does not mean complete “lysis” of sympathetic vasoconstriction but rather an attenuation that allows for increases in active skeletal muscle BF while still contributing to the maintenance of BP.

In addition to sympathetic control, non-adrenergic vasoconstrictor and vasodilatory compounds also contribute to the regulation of contracting skeletal muscle BF (Saltin and Mortensen, 2012; Holwerda et al., 2015). These can be released by skeletal muscle, endothelial cells, nerve terminals, and circulating erythrocytes in response to increased mechanical stimuli and metabolic activity during exercise. The interplay between these factors is complex and incompletely understood due, in part, to significant redundancy. Moreover, the participation of each pathway or compound may change from the onset to steady-state exercise (Clifford and Hellsten, 2004). Vasodilatory compounds include nitric oxide (NO), prostacyclin, adenosine, potassium, and ATP (Clifford and Hellsten, 2004; Clifford, 2007), which can act through endothelial-dependent or independent pathways. Additionally, non-adrenergic vasoconstrictors such as interstitial ATP, neuropeptide Y, endothelin 1, and angiotensin II also contribute to the vasoconstrictive influence during exercise (Holwerda et al., 2015); however, it should be noted that an attenuation of non-adrenergic vasoconstrictor pathways in active skeletal muscle has been reported (Brothers et al., 2006; Wray et al., 2007). Of note, the contribution of non-adrenergic vasoactive compounds to BF responses during exercise may not be as prominent in healthy individuals; however, it appears to increase with aging and disease (Schreuder et al., 2014; Barrett-O'Keefe et al., 2015; Nyberg et al., 2015).

## Neurovascular Dysregulation During Exercise in Type 2 Diabetes

**Figure 2:**
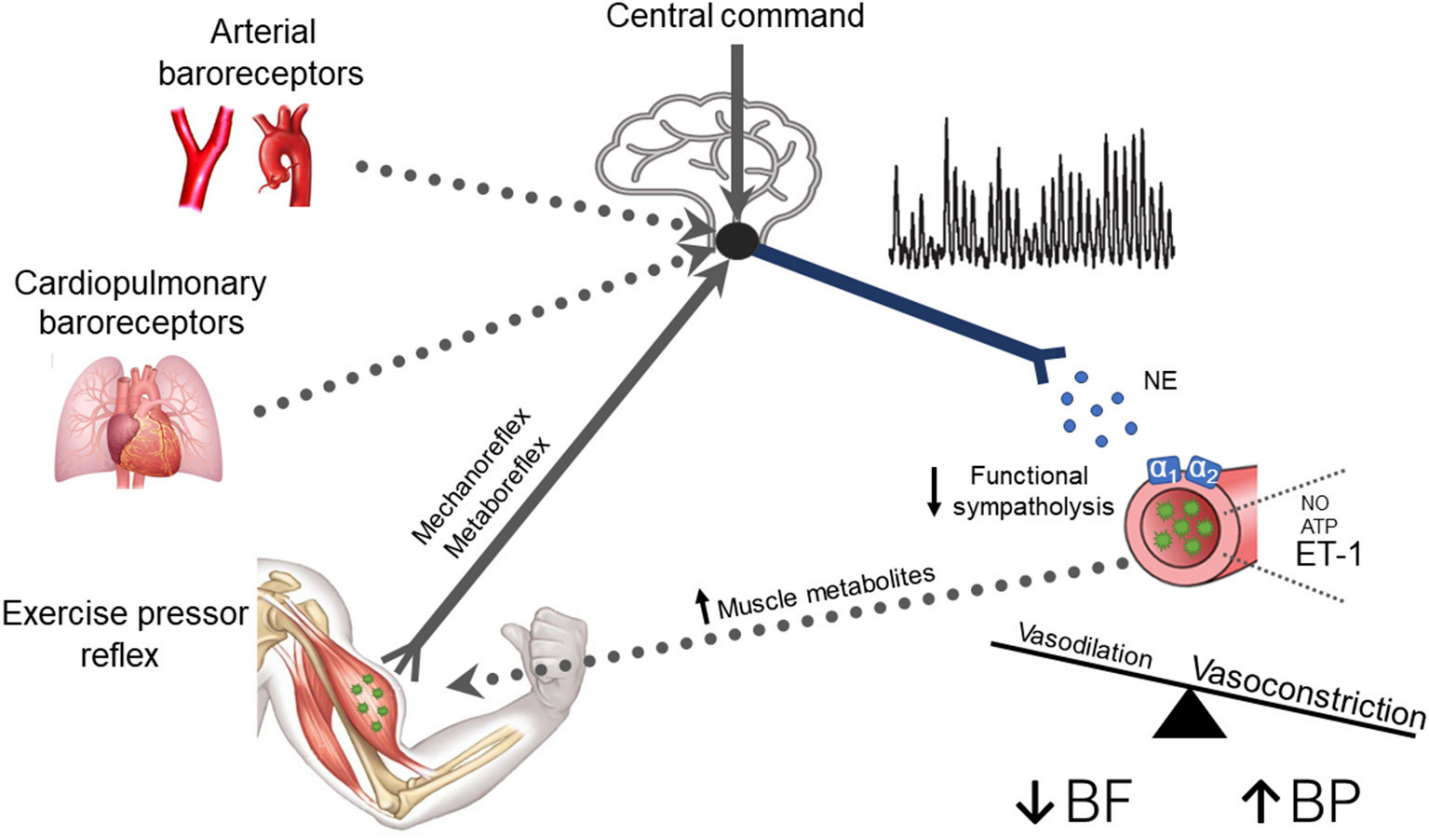
A schematic of potential neural mechanisms responsible for neurovascular dysregulation in type 2 diabetes (T2D). Current evidence supports a contribution of the exercise pressor reflex and central command (solid lines), whereas the contribution of the arterial and cardiopulmonary baroreflex remains unclear (dotted lines). Briefly, T2D patients exhibit exaggerated muscle sympathetic nerve activity (MSNA) responses, which increases the release of norepinephrine (NE) binding to α_1_ and α_2_ adrenergic receptors causing constriction of peripheral vascular beds. T2D may also impair the normal ability to attenuate sympathetically mediated vasoconstriction in active skeletal muscle (i.e., reduced functional sympatholysis). Additionally, enhanced endothelin-1-mediated vasoconstriction and reduced nitric oxide (NO) and adenosine triphosphate (ATP) mediated vasodilation may also contribute to the reduced exercise hyperemia in T2D. These alterations favor vasoconstriction, leading to reduced active skeletal muscle blood flow and exaggerated BP responses to exercise. Thus, it is plausible that T2D leads to a positive feedback scenario where enhanced MSNA and reduced functional sympatholysis combined with other impaired local vascular control pathways augment metabolic distress, which in turn further enhances MSNA by stimulating the muscle metaboreflex.

Until recently, little was known regarding the underlying mechanisms for the impaired cardiovascular responses to exercise observed in T2D. In this regard, emerging evidence suggests that the exercise pressor reflex plays a prominent role in evoking exaggerated MSNA and BP responses to exercise in T2D (Figure 2). For example, T2D rats have exaggerated renal SNA and BP responses to electrically evoked static muscle contractions in the absence of central command (Grotle et al., 2019; Kim et al., 2019). Both the mechanoreflex and metaboreflex have been shown to contribute to the exaggerated exercise pressor reflex in T2D. Indeed, T2D rats exhibit augmented BP responses to isolated mechanical stimuli (i.e., tendon stretch), suggesting an augmented mechanoreflex (Grotle et al., 2019). Furthermore, studies in humans suggest T2D also augments the metaboreflex (Holwerda et al., 2016a; Roberto et al., 2019). Specifically, Holwerda et al. (2016a) showed exaggerated MSNA and BP responses to postexercise ischemia (PEI) following isometric handgrip exercise (30 and 40% maximal voluntary contraction: MVC; Figure 1). This maneuver traps the metabolites produced during exercise and isolates the muscle metaboreflex. Interestingly, the magnitude of MSNA response to PEI was positively correlated with metabolic markers of disease severity (glucose, HbA1c, and HOMA-IR), which suggests that the severity of disease has significant impact on the enhanced expression of the exercise pressor reflex in T2D. In this regard, a recent study (Roberto et al., 2019) that reported a normal BP response to PEI following rhythmic handgrip exercise (30% MVC) had a cohort of T2D patients with relatively well-controlled blood glucose (106.41 ± 11.2 mg/dl; HbA1c 7.05 ± 0.10%). Although it is possible that the lower-intensity rhythmic exercise did not produce sufficient metabolic stimuli to unmask a difference in metaboreflex-induced BP responses between groups, notably T2D patients in this study exhibited exaggerated vasoconstriction during PEI. Interestingly, a recent study showed enhanced BP responses to ischemic rhythmic handgrip exercise (30% MVC) in nondiabetic individuals with greater insulin resistance than those with lower insulin resistance (Hotta et al., 2020). Overall, the existing literature supports that both components of the exercise pressor reflex (i.e., mechano- and metabo-reflex) may be enhanced in T2D (Figure 2).

The underlying mechanisms for an augmented exercise pressor reflex in T2D remain unknown. Specifically, it will be important to determine whether these mechanisms involve greater metabolite accumulation during skeletal muscle contraction, enhanced afferent sensitivity, or increased expression of mechanically or metabolically sensitive receptors and/or channels, or abnormal central integration of afferent feedback. It is possible that a slowed and attenuated hyperemic response to contracting skeletal muscle during exercise enhances muscle metabolite buildup, thereby increasing the activation of the metaboreflex and simultaneously sensitizing the mechanoreflex (Adreani and Kaufman, 1998; Holwerda et al., 2016a; Nesti et al., 2020). Additionally, there are reports of increased reliance on carbohydrate metabolism (Martin et al., 1995; Scheuermann-Freestone et al., 2003), reduced capillary density and recruitment, increased fast twitch muscle fiber type recruitment (Marin et al., 1994; Padilla et al., 2006; Womack et al., 2009), and increased leg lactate output during exercise (Martin et al., 1995) in T2D. These alterations may indicate a greater propensity for metabolite production during exercise in T2D. Furthermore, oxidative stress may also contribute as it is an important mediator of well-known complications of T2D (e.g., vascular dysfunction and peripheral neuropathy) (Giacco and Brownlee, 2010) and may directly influence thin fiber afferent activity (Delliaux et al., 2009). Indeed, studies have reported that reactive oxygen species may play a role in evoking the exaggerated exercise pressor reflex in common comorbidities of T2D (e.g., hypertension, peripheral artery disease, and heart failure) (Koba et al., 2009, 2013; Wang et al., 2009; Muller et al., 2012; Harms et al., 2017). Thus, it is plausible that the increased presence of metabolites and/or reactive oxygen species may contribute to the exaggerated exercise pressor reflex in T2D (Grotle and Stone, 2019). However, additional studies are needed.

Recent evidence suggests that insulin and glucose may also play a role in augmenting thin fiber afferent activity. Specifically, Hotta et al. (2019) used a whole-cell patch-clamp preparation to show that local application of insulin to small dorsal root ganglion in healthy mice decreased their mechanical threshold and augmented the amplitude of mechanically activated currents, whereas antagonizing insulin receptors attenuated this response. Furthermore, they used an isolated muscle-nerve preparation to show that application of insulin decreased the threshold, but not the magnitude, of mechanically evoked group IV afferent activity. In terms of glucose, a recent study showed that acutely infusing glucose into the hindlimb to concentrations observed in T2D rats did not affect the exercise pressor reflex or either of its two components (i.e., mechanoreflex and metaboreflex) in healthy rats (Huo et al., 2020). However, these responses may be different in T2D rats. Notably, Ishizawa et al. (2021) recently reported an augmented pressor and renal SNA response to capsaicin (chemically sensitive TRPV1 receptor agonist) administration in the hindlimb of T2D rats compared to controls. Furthermore, group IV muscle afferents isolated from T2D rats exhibited exaggerated capsaicin-induced discharge frequency, which was related to blood glucose concentrations. This aligns with findings in T2D patients showing a positive association between the MSNA response to isolated muscle metaboreflex activation and fasting blood glucose and HbA1c (Holwerda et al., 2016a). Thus, together these findings suggest that both insulin and glucose may play a role in augmenting the exercise pressor reflex in T2D. However, further studies are warranted.

Whether central command is impacted by T2D has received less attention. However, a recent study by Vranish et al. (2020) reported augmented MSNA and BP responses in T2D patients at the early onset of exercise, evident as early as 10 s into isometric handgrip. Although by no means direct evidence for central command involvement, these data at least suggest the possibility of heightened central command responses in T2D. A recent study (Kim et al., 2019) using a high-fat diet and streptozotocin-induced T2D model in rats supports a contribution of central command in evoking exaggerated SNA and BP responses to exercise (Figure 2). These investigators reported that neural stimulation of the mesencephalic locomotor region, a putative area for the central command pathway, resulted in greater renal SNA, HR, and BP responses in T2D rats compared to controls. The underlying mechanisms for these responses are not known but could involve direct alterations to brain areas responsible for central command, or the central integration of signals. Possible contributors include enhanced central angiotensin II or oxidative stress-mediated reductions in NO within the NTS, both of which have been proposed as contributors to augmented SNA reactivity to exercise in hypertension, a common comorbidity in T2D (Song et al., 1994; Zimmerman et al., 2002; Leal et al., 2012, 2013). T2D also appears to impair dynamic cerebral autoregulation during exercise, which suggests the potential for direct cerebral vascular contributions (Vianna et al., 2015). For more information on the effects of T2D on cerebral vascular regulation during exercise, the reader is directed to a recent review (Kim et al., 2020).

It is also plausible that enhanced SNA responses during exercise in T2D include an altered interaction between central command and the exercise pressor reflex (Amann et al., 2008). Painful peripheral diabetic neuropathy is a common feature in T2D and affects similar sensory afferents as those evoking the exercise pressor reflex (Davies et al., 2006). Thus, considering that effort sense influences central command (Williamson et al., 2001), it is possible that activation of sensory afferents (i.e., ergoreceptors and nociceptors) evoking augmented SNA and muscle pain also increases central command by influencing one's perceived effort during exercise. Indeed, ischemia-induced muscle pain has been shown to increase effort sense during light resistance exercise in healthy individuals (Hollander et al., 2010). Moreover, attenuating afferent feedback during dynamic exercise lowers ratings of perceived exertion during exercise, a marker for central command activation (Amann et al., 2010). Notably, there is some evidence of higher perceived effort during exercise in T2D (Huebschmann et al., 2009; Kim et al., 2015). However, these interactions are complex and require further investigation.

Whether T2D impairs arterial and cardiopulmonary baroreflex function during exercise has not been directly tested (Figure 2). Several studies indicate that T2D attenuates cardiac baroreflex sensitivity at rest (Holwerda et al., 2016b; Cseh et al., 2020; Kuck et al., 2020). However, this may not be specific to T2D, as weight-matched controls also exhibit impaired cardiac baroreflex control at rest compared to lean controls suggesting that obesity rather than T2D impairs arterial baroreflex control of HR (Holwerda et al., 2016b). In contrast, arterial baroreflex control of MSNA appears to be preserved at rest in T2D (Holwerda et al., 2016b; Moura-Tonello et al., 2016). However, whether this is true also during exercise has not been directly tested. This is important because the arterial baroreflex plays an essential role in restraining sympathetic outflow during exercise (Joyner, 2006). Likewise, to our knowledge, no study has investigated the effect of T2D on cardiopulmonary baroreflex control of MSNA (Figure 2), which also has restraining effects on MSNA responses during exercise. There is a clear need for further studies.

In addition to an exaggerated MSNA response to exercise, recent research suggests T2D also appears to impair the ability to attenuate sympathetically mediated vasoconstriction in active skeletal muscle (i.e., functional sympatholysis; Figure 2). Specifically, Bock et al. (2020) demonstrated greater vasoconstrictor responses to intra-arterial infusion of α_1_ and α_2_–adrenergic receptor agonists in active muscle during rhythmic handgrip exercise in T2D patients compared to nondiabetic controls. It is important to note that these findings contrast with Thaning et al. (2011), demonstrating preserved functional sympatholysis in T2D. However, in this study, the T2D patients were relatively healthy and had normal vasodilatory responses to acetylcholine, suggesting normal endothelial function. This is interesting because recent work (Hearon et al., 2020) suggests that the degree of functional sympatholysis may be dependent on endothelial function and thus, could, in part, explain these disparate findings.

Non-adrenergic pathways may also contribute to the reduced BF responses in active skeletal muscle reported in T2D patients. Indeed, an imbalance favoring blunted endothelial-dependent vasodilation and enhanced non-adrenergic vasoconstrictors may be involved (Figure 2) (Mather et al., 2004; Malik et al., 2005; Frisbee et al., 2019). Although limited studies have been performed, there is some evidence that warrants discussion. For example, one study (Kingwell et al., 2003) showed that attenuated leg BF responses to dynamic exercise were significantly correlated with vasodilatory responses to acetylcholine but not to sodium nitroprusside infusion, suggesting that an impaired endothelial-dependent but not independent NO-mediated vasodilation may contribute. Furthermore, impaired ATP-mediated vasodilation, perhaps due to reduced ATP bioavailability or purinergic receptor sensitivity, may also be involved (Thaning et al., 2010; Groen et al., 2019). In terms of vasoconstrictors, blocking endothelin-1 receptors during rhythmic handgrip exercise has been shown to enhance muscle BF responses in T2D patients but not in healthy controls, indicating augmented endothelin 1-mediated vasoconstriction during exercise in T2D (Schreuder et al., 2014). Collectively, these findings suggest that attenuated vasodilation via reduced NO and ATP, in combination with enhanced vasoconstrictor influence via endothelin-1, may also impair the ability to appropriately regulate contracting skeletal muscle BF in T2D. Notably, these changes in non-adrenergic pathways, along with the heightened MSNA and impaired functional sympatholysis, likely also contribute to the exaggerated BP response to exercise reported in T2D patients. Additionally, recent work suggests that augmented exercise-induced increases in arterial stiffness may also contribute to the exaggerated BP response to exercise in T2D (Cooke et al., 2020).

## Considerations and Future Directions

T2D is a multifactorial disease with many potential contributing factors to the neural vascular dysregulation reported during exercise (e.g., diet, physical inactivity, aging, family history, sex, ethnicity, comorbidities, etc.). This likely contributes to variations in diabetic phenotypes (Stidsen et al., 2018) as well as potentially differential etiologies for exercise impairments (Nesti et al., 2020), making T2D a complex population to study. It is also important to note that differences in T2D characteristics (e.g., duration of diabetes, glycemic control, and varying medications), animal models used, and exercise modality employed (e.g., type, duration, intensity of exercise) may also influence the observed neural cardiovascular responses to exercise in T2D. In addition, the majority of studies discussed in this review include relatively well-controlled T2D patients and exclude those with diabetic complications (e.g., neuropathy, uncontrolled diabetes, coronary heart disease etc.). Thus, examining neural cardiovascular responses to exercise in T2D patients with associated complications warrants future investigation. Likewise, although an augmented BP response to exercise is observed in several age groups and in both males and females, the influence of age and sex on neurovascular regulation during exercise in T2D has not been comprehensively assessed. It would be particularly interesting to determine the influence of sex hormones and menopausal status as estrogen has been shown to affect the cardiovascular responses to exercise (Schmitt and Kaufman, 2003; Fadel et al., 2004; Jarvis et al., 2011). Studies are also needed to further understand the contribution of factors such as insulin resistance, obesity, and physical inactivity. Notably, two studies indicate that insulin resistance may be a stronger predictor of an augmented metaboreflex than obesity in nondiabetic individuals (Milia et al., 2015; Hotta et al., 2020). However, others have demonstrated that obesity may drive negative effects on neural cardiovascular control mechanisms in T2D patients (Holwerda et al., 2016b). Moreover, the influence of physical inactivity on neural cardiovascular control in T2D is unclear and also warrants future investigation. Thus, overall, we have a lot more to learn about cardiovascular responses to exercise and underlying mechanisms in T2D and elucidating the role of the above mentioned factors in future studies will be important to consider as this field moves forward.

## Conclusion

T2D patients are at a twofold higher risk of premature mortality, contributing to a significantly shorter life expectancy (Raghavan et al., 2019). Emerging evidence suggests that neurovascular dysregulation leading to enhanced SNA and BP reactivity to exercise or daily physical activities (e.g., carrying groceries, walking up stairs) may increase the already heightened risk for adverse cardiac events and stroke in this population. Although mechanisms for this are just starting to emerge, evidence supports a contribution of the exercise pressor reflex and central command. However, further studies are needed and elucidation of potential roles for the arterial and cardiopulmonary baroreflex requires investigation. Additionally, augmented α_1_ and α_2_ adrenergic receptor sensitivity, endothelin-1-mediated vasoconstriction and blunted NO, and ATP-mediated vasodilation may also contribute. Indeed, T2D may lead to a scenario where enhanced MSNA, via the exercise pressor reflex and central command, along with impaired local vascular control mechanisms (adrenergic and non-adrenergic) attenuates increases in exercising muscle BF and augments the BP response to exercise. Nonetheless, there is a clear need for future studies to investigate the impact of T2D on neural and vascular control mechanisms and their interaction during exercise.

## Author Contributions

A-KG drafted the manuscript, which was critically evaluated by JK, AS, and PF. All authors approved the submitted version. All authors contributed to the article and approved the final version.

## Conflict of Interest

The authors declare that the research was conducted in the absence of any commercial or financial relationships that could be construed as a potential conflict of interest.
